# Silkworm Thermal Biology: A Review of Heat Shock Response, Heat Shock Proteins and Heat Acclimation in the Domesticated Silkworm, *Bombyx mori*

**DOI:** 10.1673/031.010.20401

**Published:** 2010-12-07

**Authors:** H. B. Manjunatha, R. K. Rajesh, H. S. Aparna

**Affiliations:** ^1^ Department of Sericulture University of Mysore, Mysore 570 006, Karnataka, India; ^2^ Department of Biochemistry, Karnatak University, Dharwad 580 003, Karnataka, India; ^3^ Department of Biotechnology, University of Mysore, Mysore 570 006, Karnataka, India

**Keywords:** acclimation, commercial traits, thermotolerance

## Abstract

Heat shock proteins (HSPs) are known to play ecological and evolutionary roles in this postgenomic era. Recent research suggests that HSPs are implicated in cardiovascular biology and disease development, proliferation and regulation of cancer cells, cell death via apoptosis, and several other key cellular functions. These activities have generated great interest amongst cell and molecular biologists, and these biologists are keen to unravel other hitherto unknown potential functions of this group of proteins. Consequently, the biological significance of HSPs has led to cloning and characterization of genes encoding HSPs in many organisms including the silkworm, *Bombyx mori* L. (Lepidoptera: Bombycidae). However, most of the past investigations in *B. mori* were confined to expression of HSPs in tissues and cell lines, whereas information on their specific functional roles in biological, physiological, and molecular processes is scarce. Naturally occurring or domesticated polyvoltines (known to be the tropical race) are more resistant to high temperatures and diseases than bi- or univoltines (temperate races). The mechanism of ecological or evolutionary modification of HSPs during the course of domestication of *B. mori* - particularly in relation to thermotolerance in geographically distinct races/strains - is still unclear. In addition, the heat shock response, thermal acclimation, and hardening have not been studied extensively in *B. mori* compared to other organisms. Towards this, recent investigations on differential expression of HSPs at various stages of development, considering the concept of the whole organism, open ample scope to evaluate their biological and commercial importance in *B. mori* which has not been addressed in any of the representative organisms studied so far. Comparatively, heat shock response among different silkworm races/strains of poly-, bi-, and univoltines varies significantly and thermotolerance increases as the larval development proceeds. Hence, this being the first review in this area, an attempt has been made to collate all available information on the heat shock response, HSPs expression, associated genes, amino acid sequences, and acquired/unacquired thermotolerance. The aim is to present this as a valuable resource for addressing the gap in knowledge and understanding evolutionary significance of HSPs between domesticated (*B. mori*) and non-domesticated insects. It is believed that the information presented here will also help researchers/breeders to design appropriate strategies for developing novel strains for the tropics.

## Introduction

In recent years, the processes of heat shock responses and the role of heat shock proteins (HSPs) have not been confined merely to ““molecular chaperons”” (Parsell and Lindquist 1991), but spread over to determine their ecological and evolutionary role in the post genomic era (Sorensen and Loeschcke 2007). It is well known that both prokaryotic and eukaryotic cells respond to unfavourable environmental conditions by increased synthesis of stress proteins such as HSPs. It is a universal phenomenon that most of the HSPs have conserved sequences ranging from bacteria to human, but certain features of the response do vary from organism to organism (Craig 1985). Unlike humans, plants and insects have a narrow range of tolerance to elevated temperatures and hence struggle to cope with these conditions. Consequently, the organisms that adapt over a period of time thrive whilst the others become extinct. For example, although *Bombyx mori* L. (Lepidoptera: Bombycidae) is derived from a wild progenitor *Bombyx mandarina* (Arunkumar et al. 2006), it has lost its temperature-tolerance due to continuous domestication over 5000 years. As a result of such a long period of domestication silkworm races/strains diverged and the strains (polyvoltines) grown in tropical environments became resistant to high temperatures and diseases, while the strains reared in temperate conditions (bivoltines) remained susceptible.

Current research points to the fact that the loss of tolerance to environmental insults in *B. mori,* unlike *B. mandarina,* is due to prolonged domestication, which offers opportunities for systematic reinvestigation of this phenomenon while substantial diversity remains among various silkworm strains/races. Concerted efforts have been made during the past two decades resulting in the evolution of heat-tolerant silkworm strains, in response to conventional breeding strategies. This process has been successful, to some degree, in the tropical environment of the Indian subcontinent. Among several breeds developed, the only bivoltine silkworm breed that performed better all through the year over three decades in the field was NB4D2. Other temperate breeds were season-dependent. This observation poses the obvious questions regarding whether or not this adaptation is due to thermal acclimation, or if it is a process of hardening. A systematic investigation followed not only with reference to NB4D2, but also other geographically distinct silkworm strains such as Diazo (p50), Nistari etc. Consequently, many qualitatively and quantitatively superior productive/robust (thermotolerant) breeds were developed using Japanese commercial hybrids (temperate origin) as genetic resource material. Due to their low tolerance to the fluctuating environmental conditions in tropical climate they become unsuitable for growing year round (Nazia et al. 2005). Thus, the efforts made in the previous three decades were futile, and the spread and success of silkworm rearing was mainly due to the introduction of F1 hybrids of native multivoltine as female parent (for resistance) and bivoltine as male parent (for high quality silk). Even now it is a challenging task to develop not only stress-and disease-resistant strains, but also to provide high yielding silkworm strains with improved stress tolerance.

The cellular stress responses were described in *Drosophila melanogaster* for the first time by Ritossa (1962) and the term ““heat shock protein”” was introduced by Tissieres et al. (1974) as these proteins increased in synthesis due to sudden increases in temperature. HSPs are identified based on their molecular mass ranges from 19 to 110 kDa in size and are broadly classified as large (major) HSPs and small HSPs (SmHSPs). The large HSPs are involved in major physiological processes such as cell division, transcription, protein folding, transport, membrane functions (Alique et al. 1994; Chen et al. 1996), and cytoprotective functions (Bakau and Horwich 1988; Chirico et al. 1988; Deshaies et al. 1988; Mizzen and Welch 1988; Palleros et al. 1991; Garrido et al. 2001; Kregel 2002). They can also form as large oligomeric complexes (Bentley et al. 1992; Leroux et al. 1997; Haslbeck et al. 1999) playing important roles in thermotolerance in mammalian cells (Landry et al. 1989), *Drosophila* (Landry and Huot 1995), house fly (Tiwari et al. 1997), and *Lucilia cuprina* (Tiwari et al. 1995) cells; but not in yeast cells (Nicholl and Quainlan 1994). SmHSPs bind specifically to cytoskeletal elements such as actin, and to intermediate filaments such as desmin, vimentin, and glial fibrillay acidic protein (Bennardine et al. 1992; Nicholl and Quainlan 1994). It has also been reported that SmHSPs modulate apoptosis (Arrigo 1998, 2005) and are involved in cell growth and differentiation (Mehlen et al. 1997). Recent approaches in genome wide identification of HSF (heat shock factor) —— targeting genes provide novel insights into the complex metabolic reprogramming that occurs in cells in response to stress (Hahn et al. 2004).

Even to date, although some information is available, applicable data are insufficient to envisage the biological importance of HSPs in *B. mori.* To understand the complex phenomena governing silkworm thermal biology, integrative genomic, proteomic, and biotechnological approaches are required. Hence, the present review aims to collate research findings accrued over the last 16 years on the heat shock response, HSPs expression, associated genes, amino acid sequences, and thermotolerance in *B. mori.* It is believed that this review will help to uncover gaps in knowledge in this area that have not been documented to date in any other organisms. We also aim to explore the possibility of using heat shock proteins as molecular markers in conventional-molecular breeding for improvement of silkworm strains.

### Heat shock and thermotolerance

The terms ‘‘heat shock’’, ‘‘acclimation’’, and ‘‘hardening’’ are commonly used to describe the changes in an organism's living state caused by external environmental conditions and treatments (Bowler 2005; Loeschcke and Sorensen 2005; Lagerspetz 2006). The usage of these terms in the silkworm thermal biology literature has not been well-defined and requires systematic study to draw a line between them. The thermal tolerance of economically important organisms to environmental fluctuations attains significance in field-rearing conditions as performance in field/nature mainly depends on native adaptability to varied environmental conditions which is governed by molecular mechanisms of the cell. Notably, the polyvoltine silkworm strains exhibit better survivability over bivoltine strains, which might be due to their adaptation to thermal stress. For example, a polyvoltine strain, C. nichi, proved to be more tolerant than the bivoltine strain NB4D2 (Joy and Gopinathan 1995). Interestingly, in India among bivoltines, NB4D2 exhibited better tolerance to environmental fluctuation both in laboratory and field conditions compared to other newer bivoltine hybrids (CSR2, CSR4, NP2, KSO1, etc.), (Vasudha et al. 2006), an observation also confirmed by the recent study of Firdose and Reddy (2009). However, the Chinese race, Feng, was the most tolerant strain followed by Japanese races, Kuo and J-09, while another Chinese race, C-54, was most susceptible (Hsieh et al. 1995). Since the range and significance of individual adaptive reactions differ in various species under different environmental conditions, the level of tolerance in elevated temperature varies between polyvoltine and bivoltine strains/races of *B. mori.* This diversity could be due to the fact that the races (species) living in hot and desert conditions for many thousands of years altered the molecular-biological mechanisms of adaptation, which facilitated their normal life-cycle even under extreme conditions (Evgen'ev et al. 2005).

Furthermore, the researchers' earlier study (Vasudha et al., 2006) demonstrated for the first time that the heat shock response in five bivoltine breeds (NB4D2, NP2, KSO1, CSR2, and CSR4) varied during different developmental stages. Of the five instars young silkworms, including first, second, and third instars, were relatively sensitive to any given heat shock temperature while older silkworms tolerated high temperatures for relatively longer periods of time. Similar observations were also reported by Joy and Gopinathan (1995). Notably, thermotolerance increased as larval development proceeded, sequentially in the order of first instar > second instar > third instar > fourth instar > fifth instar (Vasudha et al. 2006). The highest mortality (21%) was observed in the first instar and 100% survival in the fifth instar larvae of CSR4, while NP2 exhibited relatively lower mortality in the first instar and also 100% survival in the fifth instar. Comparatively, another lepidopteran model species, *Manduca sexta,* exhibited 100% survival at 42°° C, but mortality increased as the heat shock temperature was raised to 48°° C in the fifth instar (Fittingoff and Riddiford 1990). In the case of pure mysore, a tropical multivoltine strain of *B. mori* in India, no mortality occurred at 42°° C (for one hour) and 100% mortality was noticed at 46°° C (Lohmann and Riddiford 1992). Interestingly, a few Japanese and Chinese silkworm strains also exhibited 100% mortality at 46°° C after one hour of induced heat shock (Hsieh et al. 1995). In comparison with other insects, the threshold temperature that induced 100% mortality was 40°° C in *D. melanogater* (Lindquist 1986); 45°° C in different strains of silkworm, viz., NB4D2, NP2, KSO1, CSR2, and CSR4 (Vasudha et al. 2006); 46°° C in Chinese, Japanese (Hsieh et al. 1995), and Indian silkworm strains of *B. mori* (pure mysore) (Lohmann and Riddiford 1992; Joy and Gopinathan 1995); 46°° C in *Musca domestica* (Tiwari et al. 1997); 48°° C in *Manduca sexta* (Fittingoff and Riddiford 1990); 48°° C in *Lucilia cuprina* (Tiwari et al. 1995); and 50°° C in *Locusta migrotoria* (Qin et al. 2003). However, no such information is available for wild silkmoths (Tasar - *Antheraea mylitta*; Muga —— *A. assamensis*; Eri - *Samia cynthia ricini*). Hypothetically, all these studies imply that the heat shock treatment could be employed to determine the level of thermotolerance based on mortality (Loeschcke and Sorensen 2005), and they reveal that thermotolerance varies in different strains/races of silkworms and other insects.

The high thermotolerance in fifth instar larvae of *B. mori* reflects its adaptation to high temperatures that are encountered in the course of their normal life. However, in existing rearing practices, young silkworm larvae are recommended to be reared at high temperature (28°° C) and high relative humidity (RH 80%); whereas older silkworm larvae are reared at lower temperature (24°° C) and humidity (RH 65%). These practices thus leave ambiguity over the impact of heat shock on larval biological and commercial traits. In our estimation, the reason why farmers lose cocoon crops during the summer is likely due to elevated cell stress caused by high temperatures during rearing of young silkworm larvae.

### Acclimation and hardening

As pointed out above, there is a need to differentiate acclimation from heat shock responses with special reference to *B. mori.* As per Lagerspetz (2006), there are three or more definitions of thermal acclimation; and an infinite number of possible combinations can be derived between acclimation, hardening, and heat shock responses (for details see Bowler 2005; Loeschcke and Sorensen 2005; Lagerspetz 2006). Importantly, the suggested definition of acclimation, from the Commission of Thermal Physiology of the International Union of Physiological Sciences, includes hardening and heat shock (Bowler 2005; Loeschcke and Sorensen 2005). Prosser (1955) used the term acclimation for phenotypic adaptive alterations exhibited by individual organisms. A very good example of developmental plasticity, which is known as seasonal polyphenism, is that of adult butterflies belonging to the genus *Bicyclus* that exhibit different wing patterns and variation in egg size representing alternating generations between a wet season form and a dry season form (Brakefield et al. 2007). Furthermore, species and populations adapt through natural selection, operating on generations of individuals and their hereditary property. In the light of these observations, we speculate that the polyvoltine and bi-/univoltines of *B. mori* likely adapted to dry (tropical —— polyvoltine type) and wet (temperate-bivoltine type) seasons, respectively, during the course of domestication. In *B. mori,* this adaptation did not exhibit any developmental plasticity in adult phenotype beyond changes in egg sizes, which are small in polyvoltine compared to bi- and/or univoltines and diapause in bi-/univoltine eggs.

More precisely, the term acclimation may be used to describe longer-term treatments, which may or may not be beneficial, but that is dependent on the exact conditions of treatment and the trait tested (Bowler 2005; Loeschcke and Sorensen 2005). Whether this phenomenon rightly fits with the treatment given for evaluation of thermotolerant (Robust) bivoltine breeds developed utilising Japanese thermotolerant hybrids as one of the parents needs to be analysed appropriately. However, comparison of the resultant robust bivoltine hybrids (CSR18, CSR19, HT1, etc.) subjected to thermal treatment revealed more tolerance to high temperature treatments than productive breeds affecting not only the survivability, but also other cocoon traits of the insect (Suresh et al. 1999). Unfortunately, the performance of the thermotolerant bivoltine breeds under fluctuated environment was very poor in the field. Thus, the question regarding the role of stress responses in thermal adaptation in nature still remains unanswered in *B. mori* as well as other organisms with different geographical origins. Additionally, some related questions, which were asked 10 years ago, are still valid and remain unresolved. Perhaps, cross-disciplinary approaches integrating proteomic, genomic, evolutionary, biological, and physiological methods might help to address these questions.

### **Proteome approach - *expression of HSP in tissues and whole organism***

The expression, regulation, localization, and functions of heat shock proteins have been studied extensively in different organisms. The kinetics of HSP synthesis revealed distinct and reproducible differences between cell cultures of *B. mori* and the gypsy moth *Lymantria dispar.* While mulberry silkworm cells synthesize all HSP classes at temperature reaching 48°° C, the gypsy moth cells synthesize no proteins at a 40°° C and above and die under extreme conditions (Evgen'ev et al. 1987). In view of this, Evgen'ev et al. (1987) proposed to investigate whether high thermo-resistance was inherent only in the cultured cells, or if cells also behave in a similar way *in vivo.*

**Figure 1.**
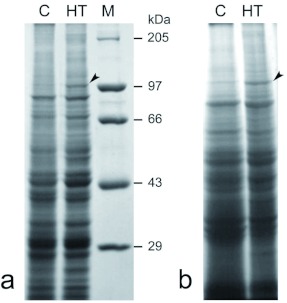
Protein profile of *Bombyx mori* larvae, heat shocked (HT) at 40°° C and untreated control (C). (A) The first instar larvae of Nistari strain; (B) The second instar larvae of P2D1 strain. Arrows denote expression of the 90 kDa heat shock protein. M indicates molecular weight marker. (Only representative images have been presented for different silkworm strains; for details refer to Vasudha et al. 2006). High quality figures are available online.

**Figure 2.**
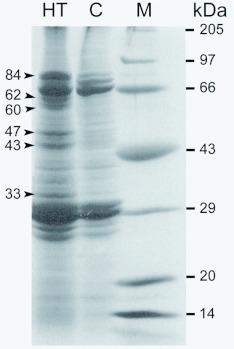
Protein profile derived from the fifth instar *Bombyx mori* larvae of CSR2 strain, heat shocked (HT) at 40°° C and untreated control (C). Arrows indicate expression of 84, 60, 62, 47, 42, and 33 kDa heat shock proteins. M indicates molecular weight marker. High quality figures are available online.

The differential expression of heat shock proteins in newly evolved bivoltine strains, NP2, KSO1, CSR2, and CSR4, was compared with that of the NB4D2 strain, which exhibited acclimation in the field over three decades (Vasudha et al. 2006). Interestingly, expression of only one set of HSPs with a molecular mass of 90 kDa in first, second (Figure 1), and third instars, and an 84 kDa HSP in the fourth instar was confirmed by Vasudha et al. (2006). Surprisingly, five different sets of 84, 62, 60, 47, and 33 kDa HSPs were also observed in the fifth instar larvae of NB4D2, KSO1, and CSR2 strains (Figure 2). Whereas, in the other two bivoltine strains expression of three HSPs (84, 47, and 33 kDa) in the NP2 and only two HSPs (84 and 47 kDa) in the CSR4 strains were reported (at 35 and 40°° C for 2 h, Vasudha et al. 2006). In a multivoltine silkworm strain, pure mysore, 84, 70, 31, 30, and 29 kDa HSPs at 42°° C (1 h, Lohmann and Riddiford 1992) and 83, 80, 74, 70, 68, 25, and 23 kDa at 48°° C were found expressed in cells and organs (for 1 h, Evgen'ev et al. 1987). Between two multivoltines, 93, 46, and 28 kDa HSPs from pure mysore and 93, 70, 46, and 28 kDa HSPs from *C. nichi* were reported (Joy and Gopinathan 1995). This clearly indicated that different sets of HSPs were being expressed at various heat shock temperatures, in different breeds of *B. mori* of which 90 and 84 kDa HSPs were ubiquitous (Table 1). In addition, expression of HSPs in different tissues varied depending on the stage of development, the temperature, and/or at which stage exposure was performed (Joy and Gopinathan 1995). Notably, concentration of HSPs and their distribution to specific sub-cellular sites is an important factor in acquisition of thermotolerance (Kampinga 1993).

Most of these studies were carried out following single dimensional electrophoresis (1-DE) and blotting techniques that made it possible to interrogate underlying mechanisms with greater certainty, albeit higher resolution of the proteins could not be achieved. To resolve these constraints in *B. mori,* advanced proteomic tools and techniques were employed, which paved the way for understanding differentiation and identification of different HSPs in the whole body of *B. mori.* A small number of protein spots were excised from the sample and separated by two-dimensional gel electrophoresis (2-DE). After analysis of the resultant mass peptide finger prints with search engine Protein prospector, they were identified as the protein HSP70 (Rajesh et al. 2008). In addition, a comparative analysis of silk gland proteins in 2-DE gels of heat shock induced and normal silkworm larvae of NB4D2 revealed discrete differences with new and over expressed protein spots (Rajesh et al. 2009). Thus, application of advanced proteome techniques proved to be a promising approach in identification of different HSPs and opened new avenues to uncover more HSPs in *B. mori.*

### Genomic approach - *hsp* genes and amino acid sequence of *B. mori*

The HSP family consists of ubiquitous proteins, which are phylogenetically conserved from bacteria to mammals and plants (Craig 1985). They have been divided into sub-families such as HSP110, HSP100, HSP90, HSP70, HSP60, HSP40, and HSP20 on the basis of their molecular weights (Nover and Scharf 1997; Gething 1998). Although, expression of HSPs has been reported from different silkworm strains (Table 1), only a few have been characterized in *B. mori.* Recently, Landais et al. (2001) characterized a cDNA encoding a 90 kDa HSP in *B. mori* and compared it with *Spodoptera frugiperda* (both lepidopteran insects). These two cDNAs encode 716 aa (amino acid) and 717 aa proteins in *B. mori* and *S. frugiperda,* respectively, with calculated molecular mass of 83 kDa which is similar to *Drosophila.* Unlike in vertebrates, *hsp90* does not contain introns and is a unique gene both in the *B. mori* and *S*. *frugiperda* genomes. Comparison of aa sequences of *B. mori* and *S. frugiperda* with that of *D. melanogaster, Homo sapiens,* and *S. cerevisiae* revealed a high percentage of similarity and phylogenetic relationships (for details see Landais et al. 2001). Apparently, extensive study is required to determine their expression at different developmental stages of different silkworm strains as the HSP90 expression is found rather in early instars than late instars (Vasudha et al. 2006) and expression of some *hsp* genes changes during development (Craig 1985). In *D. melanogaster, hsc70-4* (constitutive *hsp* gene family) was expressed at a high level in embryos, larvae, and adults, whereas the *hsc70-1* and *hsc70-2* expression was highest in adults but not detected in larvae. The *hsc70-1* was expressed at a low level while no expression of *hsc70-2* was observed in the embryo. In *Chironomus tentans, hsc70* expression was evident at all developmental stages but slightly lower in the embryo than older stages (Karouna-Renier et al. 2003).

**Table 1.**
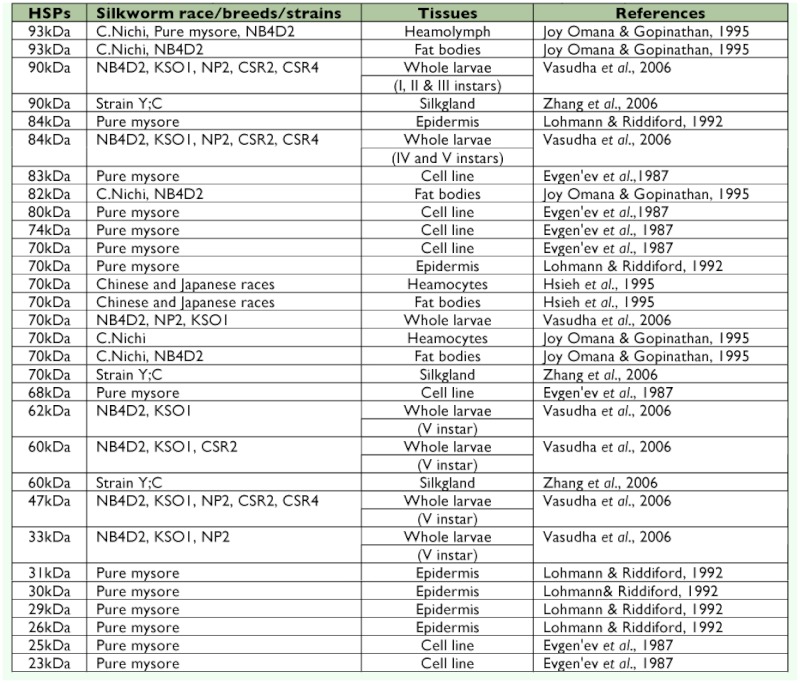
Summary of heat shock proteins expressed in different tissues and whole body of *Bombyx mori.*

Small heat shock proteins (smHSPs or sHSPs) belong to a family of genes that are seemingly less conserved compared with those of major *hsp* gene families, but occur ubiquitously in a variety of organisms. These proteins are involved in apoptosis as well as protection against heat stress (Arrigo 2005; Feder and Hofmann 1999). In *B. mori* (strain p50) six genes encoding sHSP19.9, sHSP20.1, SHSP20.4, sHSP20.8, sHSP21.4, and sHSP23.7 were reported (Sakano et al. 2006) although their biological and commercial roles remain unknown. The deduced amino acid residues of these sHSPs (Table 2) are quite similar to each other. CLUSTALW multiple alignments indicated 82, 80, and 80% identity between Pia25 and sHSP20.8, sHSP20.8, SHSP20.4, SHSP20.4, and sHSP19.9, respectively. Besides the α?-crystallin domain, the N-terminal XXLXDQXFG motifs are commonly conserved in the sequences of these HSPs (Sakano et al. 2006). Further, reverse transcriptase-polymerase chain reaction (RT-PCR) analysis showed no difference in expression levels of *smHSP* genes in different organs (Sakano et al. 2006), but indicated an increased amount of transcripts following heat shock in *B. mori* strains p50 (Sakano et al. 2006), Nistari and NB4D2 (Velu et al. 2008), which was found to be strain specific. BmHSPs (*B. mori* HSPs) with other organisms was computed using available data in National Center for Biotechnology Information (NCBI) data bank (http://www.ncbi.nlm.nih.gov) and presented in Table 3.

### Can HSP help in acquired thermotolerance?

The response to heat shock is an integral part of survival in the environment, as it is for domestic silkworms that are derived from geographically distinct regions but reared under standard conditions. Hence, a new strategy was adopted wherein the whole egg (Manjunatha et al. 2005) and larvae (Vasudha et al. 2006) of *B. mori* were subjected to heat shock at various temperatures during different developmental stages, to determine the importance of HSPs in acquired thermotolerance. These studies revealed that different sets of HSPs expressed in different developmental stages have a profound influence not only on the performance of larvae (rated in terms of mortality), but also to complete life cycle under natural environmental conditions. The well-defined role of HSPs (expressed either individually or collectively) in acquired thermotolerance in the silkworm and other insects is not known. In order to derive more accurate and novel hypotheses, the expression of heat shock proteins should be correlated with currently available information on the tolerance of silkworm strains reared in tropical environments.

**Table 2.**
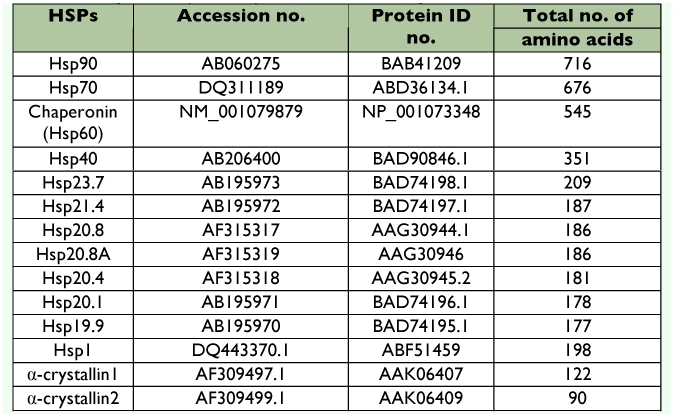
*Bombyx mori* heat shock proteins (BmHSPs) accession numbers, protein IDs and their deduced amino acids.

### Role of HSP in relation to commercial traits

To date, the greatest emphasis has been given to HSP70 and HSP90 as molecular chaperons that help organisms to cope with stresses of internal and external nature. Recent approaches not only revealed the importance of HSP90 in normal growth and development of eukaryotes, and parasite (*Plasmodium falciparum*) growth in human erythrocytes (Banumathy et al. 2003), but also elucidated the relationship between HSPs and life history traits focusing on the ecological and evolutionary relevance (Sorensen et al. 2003; Sorensen and Loeschcke 2007). Concomitantly, the relationship between heat shock, HSPs expression, and commercial traits was studied in great detail in the case of *B. mori* (Vasudha et al. 2006). Notably, an increased cocoon weight of 17.52 vs. 13.48%, and increase in shell weight of 19.44 vs. 13.45% in NB4D2 over its control was observed following heat shock at 35 and 40°° C, respectively. Concurrently, CSR2 also exhibited a 13.11 vs. 6.44% increase in cocoon weight and 16.26 vs. 5.03% increase in shell weight at 35 and 40°° C heat shock over their respective controls. The increased cocoon and shell weight observed in heat shock induced bivoltine silkworm strains compared to controls would be due to expression of HSPs at larval stage. While Joy and Gopinathan (1995) did not observe any heat shock effects on commercial traits, Lohmann and Riddiford (1992) reported that of the nine animals heat shocked at 44°° C for 1 h, only 5 resumed feeding, while 3 spun cocoons. Commercial traits of these animals were not evaluated and compared with that of controls. Consequently, as a novel strategy, heat shocked larvae (whole organisms) were allowed to grow under natural environmental conditions and they spun better quality cocoons than the non heat shocked larvae reared in natural environmental conditions (Vasudha et al. 2006). These investigations highlighted the fact that knowledge obtained from model organisms under normal laboratory conditions does not always reflect what happens out in the field, where conditions are continuously changing and unpredictably hostile. Interestingly, the increased cocoon weight and shell weight over control, reflects the positive correlation between heat shock responses and silk protein content in the cocoon. Abramova et al. (1991) reported suppression of fibroin synthesis in the silk gland following heat shock, but recently Zhang et al. (2006) identified HSP90, HSP70, and HSP60 in the silk glands of *B. mori,* offering the opportunity for further systematic investigation in different breeds of silkworm. None of the larvae recovered from heat shock at 45°° C (Vasudha et al. 2006) and 46°° C (Lohmann and Riddiford 1992), were able to spin cocoons. However, the observed differences between cocoon weight, shell weight, and shell ratio among various silkworm strains will require further investigations to determine species-specific responses to heat shock. Altogether, these observations clearly indicate that mild heat shock between 35 and 40°° C for 2 h facilitates bivoltine silkworm larvae to respond and overcome the fluctuating natural environmental conditions in succeeding instars. The practical application of this phenomenon will need to be explored positively and systematically (using multivoltine and bivoltine silkworm strains) in laboratory and field conditions in order to achieve stabilized sericulture farming in tropical countries like India.

### Hypothetical view on the heat shock and acclimation in the Silkworm, *B. mori*

Based on published scientific reports, and our own experimental observations (Manjunatha et al. 2005; Vasudha et al. 2006; Rajesh et al. 2008, 2009), it is suggested that silkworm researchers should more thoroughly delineate heat shock and thermal acclimation phenomena in *B. mori.* These studies would aid our understanding of the silkworm thermal biology much better until bio-molecular evidences further substantiate its relevance.

**Figure 3.**
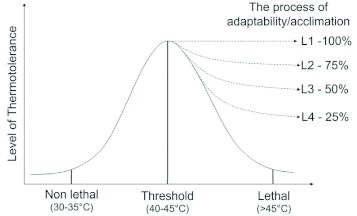
The hypothetical correlation of thermotolerance in different silkworm strains/races of *Bombyx mori* is presented. Based on the researchers' observation and other literature, non-lethal, threshold, and lethal temperatures for *B*. *mori* were determined as 30–35°° C, 40-45°° C, and >45°° C, respectively. L1, L2, L3, and L4 refer to different levels of thermotolerance, resulting in 100, 75, 50, and 25% survival rate at threshold temperatures of 40–45°° C respectively during the process of acclimation and adaptation to heat in subsequent generation. This population would be designated as thermotolerant silkworm strains suitable for tropics. High quality figures are available online.

First, the heat shock treatment given to *B. mori* larvae is to determine built in thermotolerance based on mortality and differential expression of heat shock proteins. HSP expression patterns dictate different levels of thermotolerance in individual silkworm strains at varied heat shock temperatures. The hypothetical interpretation of thermotolerance (Figure 3) refers to the state of silkworm larvae that can perform better and withstand threshold heat shock temperatures for a fixed (short) period in a particular stage or generation. Obviously, this physical state is supported by expression of one or many HSPs in a given generation, but whether the same rate of expression appears under deleterious environmental condition in subsequent generations remain unclear and will require further investigation. Thermotolerance varies among silkworm races/strains, and it is categorized by tolerance levels 1, 2, 3, and 4 (Table 3). At level-1, any polyvoltine (many generations per year, non-diapause type) or bivoltine (two generations per year, diapause at egg stage) or univoltine (one generation per year, diapause at egg stage) races/strains exhibiting better performance through several generations under fluctuating environmental conditions adapt completely. However, the survival rate varies in accordance with genomic organisation and ultimately leads to different levels of tolerance as levels 2, 3, and 4 exhibit 75%, 50%, and 25% survivability, respectively, compared to the insects that ones which did not respond to acclimation or exhibit adaptability in the natural environment. For instance, the pure mysore and nistari strains (native polyvoltines) have inbuilt adaptability to high temperatures (level 1), whereas the NB4D2 bivoltine strain (known as temperate race) exhibited better acclimation (level 2) during continuous rearing in the field round the year for three decades. As a consequence, NB4D2 acquired better adaptability than other bivoltine race/breeds/stains. Comparatively, of the new bivoltine strains, CSR2 showed better response to heat shock (Manjunatha et al. 2005; Vasudha et al. 2006) and performance in the field (level 3) than other strains (level 4) (Nazia et al. 2005). Furthermore, between polyvoltine and bivoltine strains, the Nistari exhibited higher expression of HSP70 and HSP40 genes than the NB4D2 strain (Velu et al. 2008).

Second, thermal acclimation is a longer-term treatment (ranging from days to weeks), which results in increased resistance to temperature. It is not likely to be related to HSP production as acclimation occurs within the normal temperature range experienced, and its effect lasts as long as the new acclimation conditions persist (for proposed definitions refer to Bowler 2005 and Loeschcke and Sorensen 2005). To define it more precisely in *B. mori,* the information available is insufficient and warrants further investigation in poly-, bi-, and/or univoltine strains.

Third, expression of nearly 18 different HSPs has been reported so far from various tissues and whole body of different *B. mori* strains (Table 1). Interestingly, although differential expression of HSPs is noticed during different developmental stages, their role in altering biological, physiological, and commercial traits remains enigmatic.

**Table 3.**
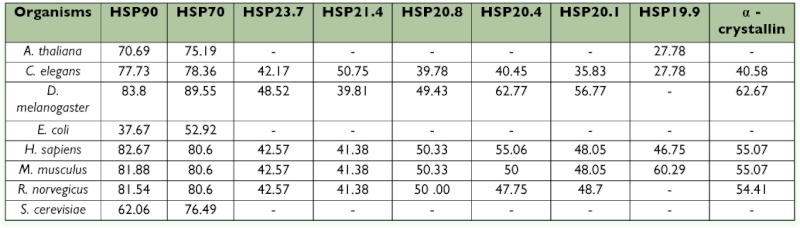
Comparative homology of *Bombyx mori* heat shock proteins (BmHSPs) with those of other organisms (%). Source : data extracted from NCBI.

In conclusion, the research resources documented here on *B. mori* would be useful for comparative genomic and proteomic research for other lepidopterans and other organisms. The genome wide analysis of *hsp* genes (Hahn et al. 2004) and their regulatory factors provide novel insights into the complex metabolic reprogramming that occurs within cells in response to stress. The domesticated silkworm *B. mori*, together with its wild progenitor, *Bombyx mandarina,* and non-mulberry silkworms (Tasar - *Antheraea mylitta*; Muga —— *A. assamensis*; Eri - *Samia cynthia ricini*), which are reared in nature, open ample scope to investigate the ecological and evolutionary modification of HSPs and identify the candidate gene(s). The individual or collective role of HSPs in relation to biological, commercial, physiological, and immunological features among different silkworm races/breeds/strains (including nonmulberry silkworms) is important for understanding the factors that govern thermotolerance and acclimation in insects. Knowledge of HSPs and their use as molecular markers would facilitate conventional breeders to select better parents, with a reduction in laborious crosses for development of suitable silkworm strains, important for tropical countries under silkworm race improvement programmes.
